# Human Induced Pluripotent Stem Cells as a Screening Platform for Drug-Induced Vascular Toxicity

**DOI:** 10.3389/fphar.2021.613837

**Published:** 2021-03-10

**Authors:** Chengyi Tu, Nathan J. Cunningham, Mao Zhang, Joseph C. Wu

**Affiliations:** ^1^ Stanford Cardiovascular Institute, Stanford University, Stanford, CA, United States; ^2^ Department of Medicine, Stanford University, Stanford, CA, United States; ^3^ Department of Radiology, Stanford University, Stanford, CA, United States

**Keywords:** vascular toxicity, IPSC disease modeling, drug testing, endothelial cells, smooth muscle cells, vascular organoids, vasculature-on-a-chip

## Abstract

Evaluation of potential vascular injury is an essential part of the safety study during pharmaceutical development. Vascular liability issues are important causes of drug termination during preclinical investigations. Currently, preclinical assessment of vascular toxicity primarily relies on the use of animal models. However, accumulating evidence indicates a significant discrepancy between animal toxicity and human toxicity, casting doubt on the clinical relevance of animal models for such safety studies. While the causes of this discrepancy are expected to be multifactorial, species differences are likely a key factor. Consequently, a human-based model is a desirable solution to this problem, which has been made possible by the advent of human induced pluripotent stem cells (iPSCs). In particular, recent advances in the field now allow the efficient generation of a variety of vascular cells (e.g., endothelial cells, smooth muscle cells, and pericytes) from iPSCs. Using these cells, different vascular models have been established, ranging from simple 2D cultures to highly sophisticated vascular organoids and microfluidic devices. Toxicity testing using these models can recapitulate key aspects of vascular pathology on molecular (e.g., secretion of proinflammatory cytokines), cellular (e.g., cell apoptosis), and in some cases, tissue (e.g., endothelium barrier dysfunction) levels. These encouraging data provide the rationale for continuing efforts in the exploration, optimization, and validation of the iPSC technology in vascular toxicology.

## Introduction

Drug-induced vascular toxicity is a multifaceted problem besetting the pharmaceutical industry, the healthcare professionals, and most importantly the patients (Qureshi et al., 2011; Herrmann, 2020). Vascular toxicity that goes unidentified during preclinical and clinical drug studies can present a serious safety hazard to the patients, often leading to drug withdrawal from the market. In fact, multiple major drug withdrawals in the past two decades are attributed to increased vascular events such as strokes and heart attacks (Qureshi et al., 2011). In addition, numerous FDA-approved life-saving chemotherapies, while effective at combating tumor growth, can also result in a wide spectrum of vascular dysfunctions, including acute vasospasm, acute thrombosis, and acceleration of atherosclerosis (Herrmann, 2020). Prevention or mitigation of these debilitating effects in patients remains a challenge for scientists and clinicians.

There is a strong need for a model that can predict drug-induced vascular toxicity, provide insights into the underlying mechanisms, and test potential therapeutics. Currently, animal models such as mice and dogs are the standard preclinical models for toxicological evaluations. Despite their indispensable role in pharmaceutical development, there is a growing recognition that existing animal models alone are inadequate for the accurate prediction of drug toxicity in humans due to differences in physiology, metabolism, and molecular functions between species (Bailey et al., 2013; Chen et al., 2013). The advent and maturation of induced pluripotent stem cell (iPSC) technology presents a valuable opportunity to solve this problem by offering a human cell model. Genetically identical to the donors, iPSCs hold the promise to recapitulate individual predisposition to various risks (Kitani et al., 2019; Lam et al., 2019). Furthermore, recent advances in iPSC differentiation strategies now enable the efficient generation of all the major cell lineages of the human cardiovascular system, including cardiomyocytes (CMs) (Lian et al., 2012; Burridge et al., 2014), endothelial cells (ECs) (Lian et al., 2014; Olmer et al., 2018; Williams and Wu, 2019; Wang et al., 2020), smooth muscle cells (SMCs) (Cheung et al., 2012; Patsch et al., 2015), and cardiac fibroblasts (CFs) (Zhang et al., 2019a). These cells functionally and structurally resemble their counterpart primary cells. Among them, human iPSC-derived cardiomyocytes (iPSC-CMs) have already shown tremendous promise in predicting chemical-induced cardiac liabilities, as exemplified by the Comprehensive *in vitro* Proarrhythmia Assay (CiPA) initiative led by the FDA (Colatsky et al., 2016; Fermini et al., 2016; Millard et al., 2018). Though similar large-scale studies have yet to be performed to validate the utility of iPSCs in vascular toxicology, emerging studies have shown encouraging results to justify further exploration of this technology as a candidate tool in preclinical and clinical investigations of drug-induced vascular toxicity. In this review, we will discuss the background of drug-induced vascular toxicity, and how iPSC technology may help us transform this field, with an emphasis on comparing the utility and drawbacks of different iPSC-based vascular models.

## Drug-Induced Vascular Toxicity

### Drug Withdrawals due to Vascular Toxicity

Unanticipated vascular toxicity is a significant cause for approved drugs to be withdrawn from the market (Qureshi et al., 2011). Using WITHDRAWN (Siramshetty et al., 2016), a publicly available database for discontinued and withdrawn drugs, we identified eight recent drug withdrawals caused by adverse vascular events. These drugs are diverse in applications, including nonsteroidal anti-inflammatory drugs (valdecoxib and rofecoxib), appetite suppressants (sibutramine, dexfenfluramine, and phenylpropanolamine), anti-diabetic drugs (benfluorex), constipation treatment (tegaserod) as well as treatment for Parkinson’s diseases (pergolide). The frequently observed risks of these drugs are strokes, myocardial infarctions (MIs), and valvular heart diseases. Among these drugs, the most recently withdrawn is sibutramine, which was found to increase the risk of non-fatal MIs by 28% and non-fatal strokes by 36% in a 2010 randomized study (James et al., 2010), and it was withdrawn from the United States market in the same year. Notably, the median time on the market of these medications, from their initial approvals to the withdrawals, is 16 years, with the longest for phenylpropanolamine (42 years) and the shortest for valdecoxib (3 years). Undoubtedly, the observed delay of these necessary withdrawals puts patients at risk and creates an extra burden to the healthcare system. Furthermore, these withdrawals highlight the inadequacy of the current system to accurately detect and predict drug-induced vascular dysfunctions.

### Vascular Toxicity by Chemotherapies

Chemotherapy drugs are widely known for having cardiotoxic effects (Orphanos et al., 2009; McGowan et al., 2017). However, they also cause a wide spectrum of vascular dysfunctions such as hypertension, ischemic events, and thromboembolism (Cameron et al., 2016; Nebigil and Désaubry, 2018; Herrmann, 2020). In fact, the majority of chemotherapies, both conventional ones (e.g., alkylating agents, antimetabolites, and anthracyclines) and targeted ones (e.g., tyrosine kinase inhibitors, proteasome inhibitors, and monoclonal antibodies) have varying degrees of vascular toxicity (Herrmann et al., 2016; Luu et al., 2018; Sayed et al., 2019; Herrmann, 2020; Rhee et al., 2020). However, unlike those drugs withdrawn from the market, the life-saving nature of chemotherapeutic agents generally tips the scale in the benefit-risk assessment, allowing them to be continuously used to treat cancer patients despite the observed risk to the vasculature. Detection and management of chemotherapy—induced vascular injuries is an important yet highly challenging task, with several major difficulties. First, there is a lack of specific circulating biomarkers for early vascular injuries in humans (Louden et al., 2006; Mikaelian et al., 2014). Second, vascular susceptibility to drugs is patient-specific, depending on numerous pre-existing conditions of an individual (Oren and Herrmann, 2018). Lastly, the mode of injury varies significantly from drug to drug (Herrmann, 2020). Collectively, these challenges call for a new vascular model that allows us to perform patient-specific testing and discover specific biomarkers for vascular injuries.

## Human iPSC-Based Vascular Model

The ideal platform to study drug toxicity should meet several key criteria. Primarily, the platform needs to faithfully recapitulate the pathological responses of the target organ on molecular, cellular, and tissue levels. From a translational standpoint, the source materials required for testing must be easily accessible and consistent in quality. Lastly, moving into the area of precision medicine, the testing platform should provide patient-specific risk prediction and facilitate the development of personalized prevention and treatment (Sayed et al., 2016; Wu et al., 2019). Conventional animal models and primary cell lines can at best partially fulfill the first two requirements, but are unlikely to meet the growing demand for personalized medicine.

**FIGURE 1:**
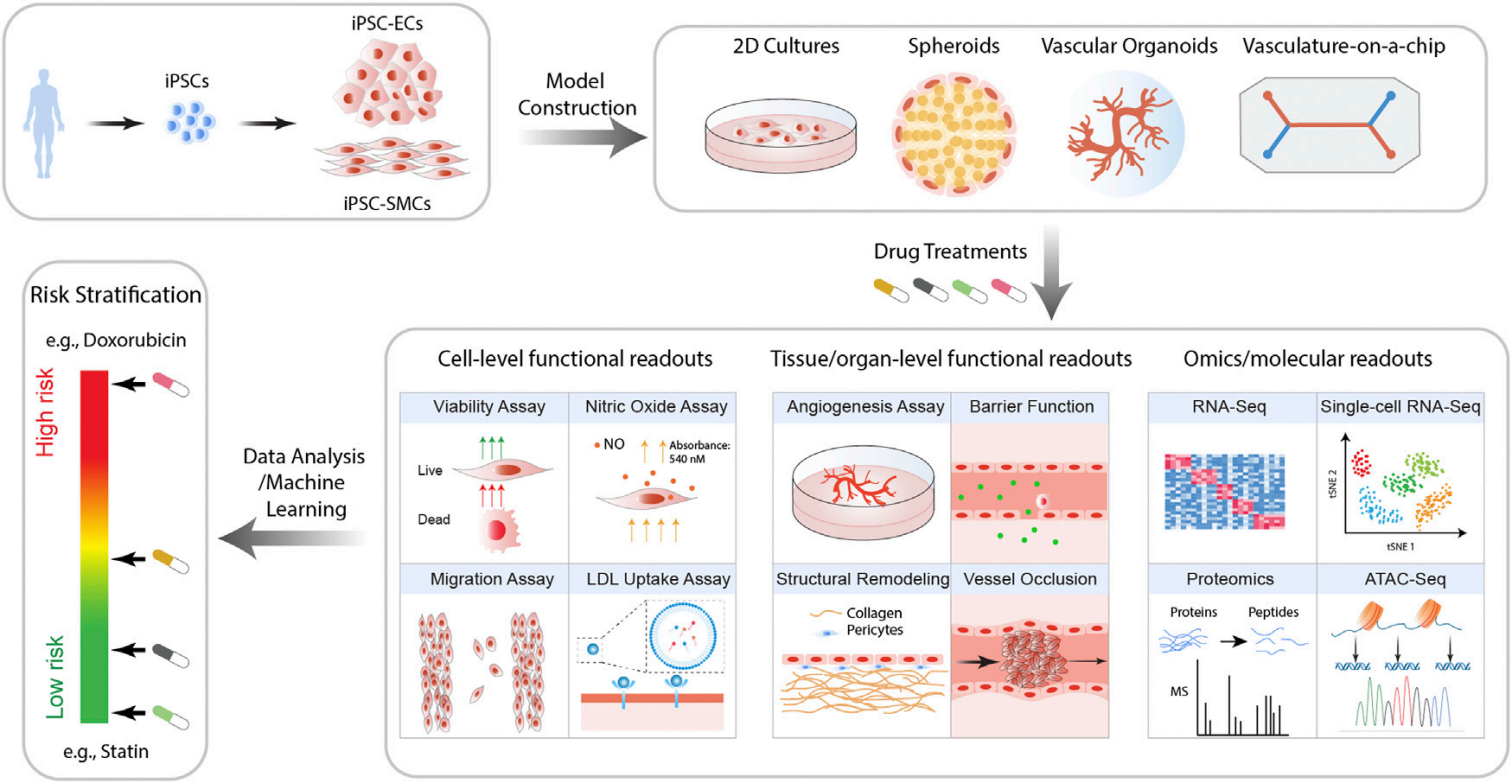
Proposed workflow for iPSC-based models for the prediction of drug-induced vascular injuries. Vascular cells such as ECs and SMCs are generated from patient iPSCs. These cells are then employed to construct *in vitro* models of varying complexity and physiological relevance, including 2D cultures, spheroids, organoids, and vasculature-on-chips. Upon treatment with drugs of interest, a wide range of functional and molecular readouts can be obtained, with which the risk of the drug for inducing vascular disorders is then calculated.

The advent of iPSC technology offers us a valuable opportunity to transform the field of toxicology. Human iPSC-derived cells are genetically identical to the donor cells and hence are expected to respond to drugs in a patient-specific manner (Burridge et al., 2016; Kitani et al., 2019). In addition, the rapid progress in iPSC technology has enabled us to efficiently generate functional ECs (Cao et al., 2013; Olmer et al., 2018; Rosa et al., 2019) and SMCs (Biel et al., 2015; Patsch et al., 2015; Granata et al., 2017), two major cell types comprising the blood vessel. Furthermore, advances in tissue engineering (Chang and Niklason, 2017; Liu et al., 2018b; Ben-Shaul et al., 2019; Crosby et al., 2019) and organ-on-chip technologies (Mori et al., 2017; Zhang et al., 2018; Wnorowski et al., 2019) have enabled us to simulate the *in vivo* vascular structure and their biophysical environment at an unprecedented pace, making it possible to model more sophisticated pathological processes on multiple scales (Figure 1).

### The Building Blocks: iPSC-Derived ECs and SMCs

EC dysfunction plays a significant role in vascular diseases such as atherosclerosis (Davignon and Ganz, 2004), thrombosis (Yau et al., 2015), and hypertension (Brandes, 2014). The generation of iPSC-derived endothelial cells (iPSC-ECs) that resemble *in vivo* ECs is a critical step toward building an *in vitro* vascular model. In the past decade, numerous methods have been developed to produce functional ECs from iPSCs, which are well summarized in a recent review (Williams and Wu, 2019). The general principle is similar among different EC differentiation methods. Human iPSCs are usually first induced into the mesoderm by a cocktail of Activin A, BMP-4, and/or GSK3 inhibitors, and subsequently specified into ECs with VEGF, FGF2, and bFGF treatment (Lian et al., 2014; Palpant et al., 2017; Liu et al., 2018a). To further enrich ECs, the differentiated cells can be sorted for the CD31 positive population (Liu et al., 2018a). In a recent study from our lab, we demonstrated that iPSC-ECs exhibit a wide range of properties characteristic of primary ECs (Sayed et al., 2020). Specifically, iPSC-ECs showed morphological similarity to primary ECs with a cobblestone cell shape. Functionally, these cells increased nitric oxide (NO) production in response to shear stress or vasodilators such as acetylcholine and A23187. Metabolically, they can incorporate acetylated low—density lipoprotein (ac-LDL), another hallmark of primary ECs.

In addition to ECs, vascular SMCs are also essential for maintaining the homeostasis of a healthy vasculature. SMCs not only provide mechanical support for a vasculature, but also regulate blood vessel tone, inflammation, and vascular remodeling (Majesky et al., 2011; Lim and Park, 2014). SMC disorder is a major contributing factor to the development of common vascular diseases such as atherosclerosis (Bennett et al., 2016) and hypertension (Touyz et al., 2018). Early methods to generate SMCs from iPSCs relied on unguided differentiation to produce a highly heterogeneous population, followed by flow cytometry sorting for Flk1^+^/VE-cadherin^-^ cells. These cells exhibited typical SMC morphology and expressed specific markers such as alpha-smooth muscle actin (αSMA) and calponin (CNN1) (Taura et al., 2009). Since then, SMC differentiation has evolved to be more efficient and targeted (Stephenson et al., 2020). For instance, Cheung et al. developed a set of protocols to produce lineage—specific vascular SMCs (Cheung et al., 2012; Cheung et al., 2014). These iPSC-SMCs not only express markers such as CNN1 and myosin heavy chain 11 (MYH11), but also differentially respond to cytokine stimulation depending on their lineages (Cheung et al., 2012). In addition to lineage specificity, Wanjare et al. reported that modulation of serum level and PDGF-BB could generate phenotype-specific (synthetic or contractile) SMCs from iPSCs (Wanjare et al., 2013). In this section, we will discuss the use of iPSC-ECs and iPSC-SMCs to model vascular injuries (Table 1).

**TABLE 1:** Recent human iPSC-based models of vascular injuries.

Year	Model	Toxicants/Stress	Main outcome	Ref
2015	2D iPSC-SMC	TNFα	Increased CX3CL1 and MMP9 expression	Biel et al. (2015)
2016	2D sprouting assay of iPSC-EC	A panel of 38 putative vascular disrupting drugs from ToxCast library	Reduced iPSC-EC sprouting and/or reduced cell viability	Belair et al. (2016)
2017	2D lineage-specific iPSC-SMC	Genetic stress: *FBN1* mutation	Reduced vascular SMC proliferation and reduced contractile stress. Increased TGF-β signaling and matrix remodeling. Identification of P38, AGTR1 and KLF4 as therapeutic targets	Granata et al. (2017)
2017	2D high-throughput iPSC-EC and HUVEC model	Suramin, nocodazole, colchicine, histamine, concanamycin A, SU5402	Reduction in nuclear content, viability, ATP content as well as angiogenesis capacity *in vitro*	Iwata et al. (2017)
2017	3D microfluidic iPSC-SMC	Genetic stress: progerin	Increased media wall thickness, increased calcification, and increased cell apoptosis	Atchison et al. (2017)
2017	3D microfluidic iPSC-EC	Thrombin and sunitinib	Disruption of cell-cell junctions in response to thrombin. Reduction in blood vessel area by sunitinib	Kurokawa et al. (2017)
2017	2D high-throughput iPSC-EC model	TKIs	Reduced viability in a dose-responsive manner across multiple cell lines	Sharma et al. (2017)
2019	3D vascular organoids with ECs + pericytes	Hyperglycemia and cytokines	Thickening of vascular basement membrane. Increased expression of collagen type IV and other basement membrane components	Wimmer et al. (2019)
2019	3D microfluidic microvessel of blood-brain barrier	TNFα	Increased expression of ICAM-1 and VCAM-1 and leukocytes adhesion	Linville et al. (2019)
2019	2D iPSC-EC	Genetic stress: ApoE (apolipoprotein) allele epsilon 4 expression	Activation of the proinflammatory state and prothrombotic state. Increased VWF expression and increased platelets adhesion	Rieker et al. (2019)
2019	2D iPSC-EC	E-cigarettes liquid	Increased cell apoptosis, ROS level and LDL uptake. Reduced migration. Conditioned medium increased macrophage activation	Lee et al. (2019)
2019	3D microfluidic blood brain barrier	TNFα, IL-1β, and IL-8	Compromised tight junctions in the barrier and increased leaking	Vatine et al. (2019)
2020	2D iPSC-EC	Cigarette smoke	Secretion of cytokines involved in coagulation, inflammation, and fibrosis	Chu et al. (2020)
2020	2D microfluidic iPSC-SMC	Genetic stress: progerin	Upregulation of MMP13 and increased detachment of SMCs	Pitrez et al. (2020)

Abbreviations: AGTR1, angiotensin II receptor type 1; CX3CL, C-X3-C motif chemokine ligand 1; ICAM-1, intercellular adhesion molecule 1; IL-1 β, interleukin 1beta. IL-8, interleukin 8; KLF4, Kruppel like factor 4; LDL, low-density lipoproteins; MMP13, matrix metallopeptidase 13; MMP9, matrix metallopeptidase 9; P38, p38 mitogen activated protein kinase; ROS, reactive oxygen species; TGF-β, transforming growth factor beta; TKIs, tyrosine kinase inhibitors; TNFα, tumor necrosis factor alpha; VCAM-1, vascular cell adhesion protein 1; vWF, von Willebrand factor.

### 2D Culture Model

To test the toxicity of a compound, the conventional approach is to apply it directly to a monolayer culture of target cells. Although they lack the sophisticated tissue architecture or biophysical stimulation (e.g., shear stress) present *in vivo*, numerous studies have demonstrated that iPSC-ECs or iPSC-SMCs cultured in monolayers respond to chemical or genetic stress in ways that are largely consistent with clinical observations or data from primary cells (Zhang et al., 2011; Cheung et al., 2012; Biel et al., 2015; Belair et al., 2016; Tang et al., 2017; Rieker et al., 2019; Chu et al., 2020). For instance, our lab recently found that e-cigarette liquid (e-liquid) triggered a broad array of molecular and functional disorders in iPSC-ECs, including activation of cell apoptosis, increased reactive oxygen species (ROS) production and LDL uptake, as well as reduced cell migration (Lee et al., 2019). Conditioned medium from e-liquid-treated iPSC-ECs induced macrophage polarization, suggesting a pro-inflammation effect of e-cigarettes on the cardiovascular system. Importantly, these data were corroborated by an analysis of the serum from e-cigarettes users, which revealed a similar upregulation of inflammatory cytokines (Lee et al., 2019).

A key advantage of a 2D model is the compatibility with high-throughput assays. Furthermore, the unlimited proliferative potential of iPSCs allows us to generate large quantities of cells required for large-scale screening experiments, which can be challenging when using primary cells. In fact, several studies have employed high-throughput platforms to investigate drug-induced vascular toxicity or to identify therapeutic agents using iPSC-ECs (Iwata et al., 2017; Sharma et al., 2017) or iPSC-SMCs (Zhang et al., 2019b). For instance, Iwata et al. used high-content imaging in a 384-well plate format to test a variety of compounds with or without vascular toxicity on iPSC-ECs (Iwata et al., 2017). Based on readouts including ATP content, nuclear content, cell viability, and tube formation, this platform successfully identified the toxic compounds with high reproducibility (Iwata et al., 2017). Moreover, the high-throughput format is also suitable for screening therapeutic compounds that could induce a desirable phenotype. Zhang et al. engineered a MYH11 reporter cell line in human embryonic stem cells (Zhang et al., 2019b). MYH11 expression was chosen as a surrogate for the desired contractile phenotype in SMCs. By doing so, they successfully identified RepSox, a small molecule inhibitor of TGF-β type 1 receptor, as a potent drug that could mitigate intimal hyperplasia, a condition characterized by the pathological switch of SMCs from the contractile to the synthetic phenotype.

### Vascular Spheroids and Organoids

Application of 3D culture is a common strategy to improve the physiological relevance of an *in vitro* model (Duval et al., 2017). Generally speaking, cells grown as 3D constructs experience more cell-cell interactions and cell-extracellular matrix (ECM) interactions than in 2D (Duval et al., 2017). In addition, a compact 3D environment creates diffusion gradients for biochemical signals, increasing the retention of secreted factors and allowing for a localized build-up of metabolic waste, all of which are typical features of the *in vivo* environment. Thanks to these advantages, 3D vascular models have been broadly exploited to investigate tumor angiogenesis and to test anti-angiogenic compounds (Taubenberger et al., 2016; Amann et al., 2017; Chiew et al., 2017; Klimkiewicz et al., 2017).

A simple method to generate vascular spheroids is to aggregate vascular cells, either stem cell-derived or primary, directly into 3D spheres. This can be done using the hanging-drop technique (Markou et al., 2020) or using an ultra—low attachment culture substrate (Moldovan et al., 2017). Interestingly, distinct cell types mixed together to form spheroids tend to spontaneously organize themselves into a structural hierarchy reminiscent of a native vasculature. For instance, Markou et al. generated small vascular spheroids (∼100 μm) from iPSC-ECs and iPSC-SMCs (Markou et al., 2020). The iPSC-ECs spontaneously formed the outer lining of these spheroids, allowing them to be in direct contact with the culture medium, whereas the iPSC-SMCs lined beneath the iPSC-ECs, forming the interior layer (Markou et al., 2020). Despite being a relatively crude model, these spheroids emulate some aspects of vascular anatomy that are not present in a 2D culture.

To create more sophisticated vascular organoids, biomaterials such as collagen, fibrin, and hyaluronic acid can be employed to initiate angiogenesis inside the organoids (Crosby et al., 2019; Crosby and Zoldan, 2019). Compared with cell—only spheroids, the introduction of biomaterials improves the control over the microenvironment, making it possible to develop high-fidelity vasculatures. This is well illustrated recently by Wimmer et al., who combined the use of fine-tuned iPSC vascular differentiation and a 3D matrix (collagen and Matrigel) to produce highly elaborate capillary vasculatures composed of ECs, pericytes, and basement membrane (Wimmer et al., 2019). These engineered microvessels have a hollow lumen and could integrate into the native vasculature upon transplantation *in vivo*, indicating the functionality of these capillaries. Upon exposure to diabetic stress consisting of hyperglycemia, tumor necrosis factor (TNF), and interleukin-6 (IL-6), these capillaries underwent massive thickening in the basement membrane as evidenced by collagen IV staining, a hallmark of diabetes-induced vasculopathy. Notably, identical treatment applied to iPSC-ECs or iPSC-SMCs in 2D cultures did not result in any increase in collagen IV accumulation, suggesting the necessity of the 3D structure to recapitulate this pathological change.

### Microfluidic Vascular Chips

Microfluidic organ-on-a-chip technology is a fast-growing field arising from the convergence of engineering, material science, and human biology. Essentially, these microchips are miniaturized physiological systems engineered to emulate key features of native organ architecture and function (Atchison et al., 2017; Kurokawa et al., 2017; Gold et al., 2019). By modulating channel geometry, substrate stiffness, perfusion rate, and biochemical signals, microfluidic chips can model a wide range of vascular diseases on an organ scale. For instance, vascular chips can be engineered with inward protrusions in the middle of the channels to mimic the narrowing of arteries caused by atherosclerosis plaque (Westein et al., 2013). This special geometrical design resulted in platelet aggregation and increased vWF expression, in alignment with the increased thrombus events observed in animals and patients with atherosclerosis. Another example by Qiu et al. showed that the perfusion of microvascular chips *in vitro* using red blood cells (RBCs) harvested from sickle patients could cause vessel occlusions in the chip, similar to the vascular obstructions seen in sickle cell anemia patients (Qiu et al., 2018).

The fusion of iPSC technology and microfluidics is revealing even more exciting possibilities. Specifically, early vascular chips mainly relied on primary ECs like human umbilical vein endothelial cells (HUVECs) to generate a functional endothelium. While these HUVEC-based models are useful as a generic representation of vasculatures, they are not ideal models for organ-specific blood vessels. In fact, it is well-known that ECs in distinct organs differ in both molecular signature and function (Marcu et al., 2018; Paik et al., 2020b). By contrast, human iPSCs can be differentiated into organ-specific ECs, allowing us to manufacture more specialized and physiologically representative vasculatures. The blood-brain barrier (BBB), for instance, is one of the most specialized and clinically important vasculatures. Researchers have demonstrated that iPSC-derived brain microvascular ECs (iPSC-BMECs) can form a functional endothelium barrier in microfluidics and exhibit a large transendothelial electrical resistance (TEER) characteristic of the human BBB (Linville et al., 2019; Vatine et al., 2019). Furthermore, the engineered BBB exhibited selective permeability and remained functional while being perfused with human whole blood (Westein et al., 2013). In addition to modeling endothelium, microfluidic platforms can also be utilized to investigate vascular smooth muscle pathology in a patient-specific manner. Using microfluidics, iPSC-SMCs derived from progeroid patients were found to be more susceptible to detachment under flow-induced shear stress compared to the healthy control, and metalloprotease 13 (MMP13) was identified as a potential therapeutic target (Pitrez et al., 2020).

### Considerations for Choosing the Model

Generally speaking, there are two fundamental considerations in choosing *in vitro* models for vascular toxicity studies or toxicity studies in general. The first consideration is physiological relevance, which determines whether a model can precisely recapitulate the human response to a given drug. Overall, organoids and microfluidic vascular chips are expected to be more physiologically relevant than 2D models or simple vascular spheroids (Duval et al., 2017). The second factor to consider is experimental throughput. This factor is especially important during the early stage of drug research and development, when it is often necessary to screen tens of thousands of compounds. 2D models, despite their limited physiological relevance, are most commonly used for high-throughput screenings to identify toxic vs. therapeutic compounds. Therefore, a rational strategy would be to employ 2D models for early exploratory investigations that are focused on narrowing down the list of candidates or gaining preliminary insights. Afterward, 3D organoids or microfluidic platforms can be adopted to develop a more comprehensive and accurate understanding of the drug’s effects.

## Moving Forward-What is Next?

### Improving the Quality of the Cells

A universal concern regarding iPSC-derived cells is that they tend to be very heterogeneous, and this is no exception for iPSC-derived vascular cells (Karakikes et al., 2015; Paik et al., 2018; Kwong et al., 2019; Volpato and Webber, 2020). Drug testing using heterogeneous cell populations may lead to an inaccurate conclusion, especially if it is based on ensemble measurements (Altschuler and Wu, 2010). To tackle this issue, a variety of strategies can be employed, such as optimization of the differentiation protocol (Wang et al., 2020), purification using genetically engineered reporter cell lines (Hu et al., 2018), or magnetic-activated cell sorting (MACS) based on the desired surface marker (Li et al., 2017).

Future iPSC-based vascular models may benefit from using tissue-specific or vessel-specific cells to improve their physiological relevance. In recent years, it is increasingly appreciated that ECs or SMCs may vary considerably in both function and molecular signature depending on their subtype (Cheung et al., 2012; Bennett et al., 2016; Williams and Wu, 2019; Jambusaria et al., 2020). Some successful efforts have been made on this front, such as the development of brain microvascular chips (Linville et al., 2019; Vatine et al., 2019; Weaver and Valentin, 2019) and drug testing using lineage-specific SMCs (Cheung et al., 2012).

### Validation Studies

One representative iPSC technology at the forefront of transforming drug toxicology is the use of iPSC-derived cardiomyocytes (Gintant et al., 2019; Pang et al., 2019). In particular, large-scale efforts exemplified by the CiPA initiative have been coordinated by academia, industry, and regulatory bodies to explore and validate iPSC-CMs as a viable option for predicting cardiac liabilities in a translational setting (Colatsky et al., 2016; Blinova et al., 2017). Clearly, for iPSC-based vascular models to be accepted as a tool in preclinical or even clinical investigation, a similar pathway is inevitable. Particularly, iPSC vascular models should be rigorously tested with double-blinded experiments to quantify the specificity and sensitivity, and their performance needs to be compared with animal models.

### Exploring Emerging Technologies

The past decade has witnessed a bountiful rise of exciting technologies in the biomedical field. These technologies can be exploited to empower iPSC-based drug testing. 3D bioprinting technologies using iPSC-derived cells can help us build more realistic vasculatures on a chip (Kolesky et al., 2014; Maiullari et al., 2018). Single-cell technologies such as single-cell RNA sequencing can reveal hidden pathological changes at an unprecedented resolution (Chaudhry et al., 2019; Paik et al., 2020a). CRISPR-based gene editing tools together with patient-specific iPSCs may unveil the effects of single nucleotide polymorphism (SNP) on drug response and hence determine individual predisposition to toxicity (Pang et al., 2009; Burridge et al., 2016; Seeger et al., 2017; Garg et al., 2018; Ma et al., 2018). Machine learning supported with big data has also shown promise in improving the prediction of drug toxicity (Lau et al., 2019; Vo et al., 2020). Collectively, these technologies have led us to uncharted territory for toxicology. The utility of iPSC-based platforms in predicting vascular toxicity, or any drug-induced toxicity, is likely to be significantly expanded in combination with these state-of-the-art tools.

## Concluding Remarks

Prediction and understanding of drug-induced toxicity is a critical part of pharmaceutical development as well as patient care. The vascular system as the network for transporting nutrients and hormones is at a high risk of exposure to drug-induced off-target damages, as evidenced by notable major drug withdrawals caused by vascular events and frequent vascular complications observed in patients undergoing chemotherapy. However, the detection of drug-induced vascular toxicity in humans is difficult, partly because its manifestation may be slow and patient-specific (Cameron et al., 2016; Herrmann, 2020). In fact, it was suggested that surveillance of vascular health in certain cancer survivors should last several years after chemotherapy (Herrmann, 2020). Obviously, such a long time frame would be impractical in most translational and clinical settings. The use of animal models is intended to accelerate the discovery and understanding of drug toxicity. However, it is increasingly recognized that fundamental species differences on molecular, cellular, and tissue levels limit the predictive power of animal models for human toxicity (Bailey et al., 2013; Bailey et al., 2014). The invention of iPSC technology presents a paradigm shift improvement in our ability to predict and dissect drug-induced vascular injuries. For the first time, it is possible to establish a scalable and personalized drug testing platform. To be sure, iPSC models are still very immature for the time being, and issues such as consistency and physiological relevance are yet to be comprehensively examined. Nevertheless, with the rapid and continuous improvements made by researchers from different disciplines, we are optimistic that they will be an increasingly powerful tool in the future of vascular toxicology.
